# Design of an automatic titrator

**DOI:** 10.1155/S1463924687000270

**Published:** 1987

**Authors:** D. Migneault, R. K. Forcé

**Affiliations:** 1 University of Rhode Island Chemistry Department Kingston Rhode island 02881 USA; 2 The Connecticut Agricultural Research Station New Haven Connecticut 06504 USA


Journal of Automatic Chemistry/*Journal of Clinical Laboratory Automation*, Vol. 9, No. 3 (July–September 1987), pp. 125–128

### Design of an automatic titrator

**D. Migneault* and R. K. Forcé†**

*University of Rhode Island*, Chemistry Department, Kingston, Rhode island 02881, USA*

*An inexpensive microcomputer-based system has been developed to conduct potentiometric titrations. A PET 2001 microcomputer is interfaced with three electronic stages to provide two addressable, readable channels, controllable stirring, and program-controlled titrant delivery. The easily altered software allows much versatility in the experimental approach. The use of a sample algorithm to conduct titrations is illustrated.*

### Introduction

Microcomputers and microprocessors in the analytical laboratory have altered the very nature of the experimental process. Instrumental control, data collection, and real-time decision-making can all be accomplished by microcomputer. Experiments can be conducted more efficiently, more precisely, and more routinely. Data storage for later analysis and/or transfer to other processors is facilitated by these systems.

Our research involves the determination of stability constants of metal ligand complexes in marine systems based on the experimental approach developed by Bjerrum [1]. The titrations necessary to determine these constants properly require precise and accurate measurements recorded during experiments of considerable duration. After attempting these experiments manually, the advantages that microcomputer control could bring to the experimentation were recognized: consistent electrode stability criteria could be imposed, constant sampling times could be rigidly maintained, and the amount of titrant to be delivered can be calculated during the experimental process and delivered accurately and precisely. Generally stated, experimental conditions could be more easily standardized. Because the software is modular, it can be easily modified to conduct a variety of potentiometric experiments. An additional advantage is that the data files can be managed much more efficiently.

Automatic titrators have advanced into the stage of having computer control and data acquisition [2 and 3]. Under computer control it has been easier to alter titration parameters [4], to use more sophistocated algorithms to conduct the titrations [4–7] and to treat the data with more advanced mathematical processes [5 and 6]. The added versatility provided by computer control allows these systems to be used in a number of similar experiments, such as spectrophotometric and amperometric titrations.

Titrators using the same 6502 central processing unit and hence the same instruction set have been constructed previously [9 and 10]. A 6502-based Rockwell Aim has been used in conjunction with the AD574JD (Analog Devices) 12 bit analogue-to-digital conversion chip [9] and a CBM type 2016 microcomputer both have been developed into titrators.

An important advantage provided by computerization of potentiometric experimentation is the ability to analyse the electrochemical data quickly and efficiently. In addition, more complicated algorithms can be applied during titration and data analysis. Consequently, a concomitant increase has occurred in the sophistication of data treatment. Methods of end-point detection and of statistical determinations of stability constants have been advanced. The linearization of the sigmoidal acid-base titration curve by the Gran plot [11 and 12] was of prime importance. This approach has been applied in several programs used by automatic titrators, and in many cases the technique and theory has been refined [13 and 14]. A number of computer-assisted methods for the calculation of stability constants by various fitting procedures have been developed [15 and 16]. More recently, these fitting programs have also been used to refine the calibration of the sensing probes themselves [17].

A simple and inexpensive system for potentiometric experimentation is reported here. It has the advantages of (1) using a commercially available microcomputer; (2) costing only a few hundred dollars in hardware; (3) having electronics which are easily fabricated; (4) providing total flexibility over all experimental parameters through BASIC; (5) allowing any titration algorithm to be utilized to conduct the titration; and (6) possessing excellent data storage and treatment capabilities.

### Instrumentation

The microcomputer we employ is a PET 2001-8 with a 4040 Dual Floppy Disk, and a 2022 Tractor Printer (Commodore Business Machines, Inc., 901 California Avenue, Palo Alto, California 94304, USA). The PET is equipped with a 16 Kbyte expansion board (Skyles Electric Works, 231 E. South Whisman Road, Mountain View, California 94041, USA). The parallel user port on the PET consists of an eight-bit data bus and a unidirectional control line CB2. The lines of the bus are individually programmable as inputs or outputs, and the bus is addressable in either assembly or BASIC. The PET is connected through the IEEE-488 port to a parallel to

* Present address: The Connecticut Agricultural Research Station, New Haven, Connecticut 06504, USA

† Correspondence to Dr Forcé.

serial conversion unit (TNW2000, TNW Corp., 3444 Hancock Street, San Diego, California 92110, USA) to a Data General Eclipse S/130.

The two electronic stages of our system, along with another analogue-to-digital conversion stage, complete the automatic titrator. Figure 1 schematically illustrates the system. The first stage is for signal conditioning. It gains, offsets, and filters electrode responses for input into an analogue to-digital (A/D) stage. The PET outputs signals from the parallel users' port to activate a motor-control stage which drives a stepper motor for

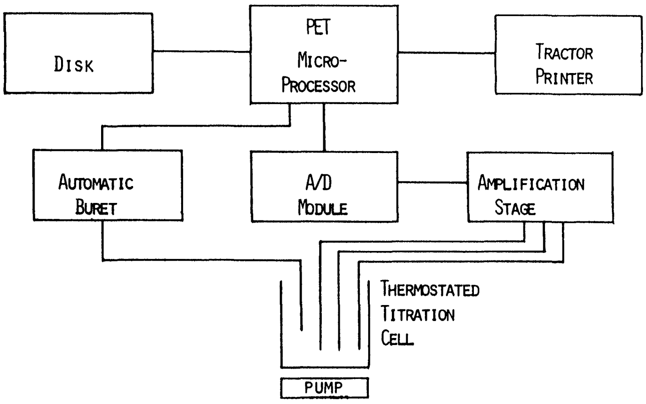

*Figure 1. Schematic diagram of the automatic titrator.*

titrant addition. In addition, the PET controls AC switching for the magnetic stirrers. An analogue-to-digital conversion module described elsewhere [18], converts the voltage into a 12-bit digital word for input into the parallel user port of the PET.

One channel of the electronics of the dual channel amplification stage is shown in figure 2. The electrode potential is transmitted to the module by means of a shielded cable, which, together with the Faraday cage constructed about the reaction vessel, minimizes noise pick-up by the high impedance electrodes. The activity-dependent potential is connected first to a high-impedance operational amplifier, UAI in the amplification stage. The gain on the amplifier is controlled by a 0–100KΩ 10-turn potentiometer which gives an amplification of up to 10X. The output voltage of OAI is summed with a variable 0–10 voltage from a divider into the second operational amplifier, OA2. The variable gain and offset serve to amplify and offset the electrode potential into the 0 to +10 volt range of the A/D module, thereby maximizing the precision of the 12 bit conversion. Three RC circuits are included on the second amplifier to filter out noise, especially 60 Hertz interference picked up by the high-impedance electrodes.

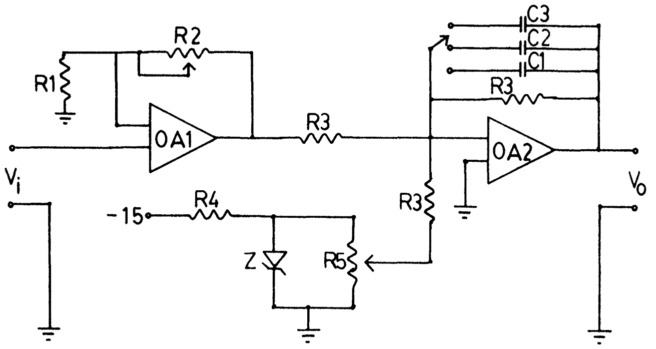

*Figure 2. One channel of the amplification stage; where R1 = 10kΩ; R2 = 100kΩ, 1 turn Potentiometer; R3 = 49.9K Ω, 1%; R4 = 460 Ω; R5 = 5K Ω, 10 turn potentiometer; C1 = 5μf; C2 = 10μf; C3 = 20μf; OA1 =μAF356 operational amplifier (Fairchild); OA2 = μA741 operational amplifier (Fairchild); Z = 10V Zener Diode.*

When activated the A/D module multiplexes one of the eight inputs and samples and holds the voltage. The module then converts this voltage into a 12-bit digital word which is output to the PET in two transfer steps through the 8-bit data bus. Software routines recombine these two bytes into the appropriate voltage reading. The read process takes 50 milliseconds. The A/D module is activated by a high to low transition on CB2 the control line during a read cycle. The stepper motor and stirrer control are turned off by this transition. Otherwise spurious communication signals between the PET and the A/D could inadvertently pulse the stepper motor in either direction.

The titrant delivery and stirring control module is schematically described in figure 3. To add titrant to the reaction vessel the stepper motor must be pulsed in the proper four-step sequence. Signals are output from the PET through data lines PA4-7 to four optoisolators. These serve to isolate the 0-5 V TTL signals used by the digital electronics from the +12 V supply used to drive the windings of the motor. The optoisolators provide a base current for power transistors which provide proper pulses for the stepper motor (North American Philips Controls Corp., Cheshire, Connecticut, USA). Short-time delays between the pulses are added in order to allow the stepper motor time to respond correctly to maintain precision of the titrant delivery. An additional output line of the data bus, PA3, is connected through a similar optoisolator to a power transistor which, in turn, provides the base current for a TRIAC, a solid-state AC switching device (Archer Electronic Parts, Tandy Corporation, Fort Worth, Texas 76102, USA). This TRIAC, by means of the base current provides 115 V AC switching. Thus, the software can have control over the stirring of the reaction solution. In a reaction vessel of a previously published design [19], the stirrer is used to drive a glass and Teflon pump motor which circulates the solution through a UV-VIS spectrophotometer. The PET computer has been interfaced with the CARY Model 210 spectrophotometer [20]. This allows for simultaneous automatic acquisition of potentiometric and UV-VIS absorbance data.

The schematic for the stepper motor and syringe burette is shown in figure 4. The syringe burette (S1200, Gilmont Instruments Inc., 401 Great Neck Road, Great Neck, New York 11021, USA) is held securely to the aluminum base plate by part of a test-tube clamp. The end of the syringe burette is connected securely to the stepper motor by means of a PVC rod drilled and tapped with set screws to secure the end of the burette in place. The motor is

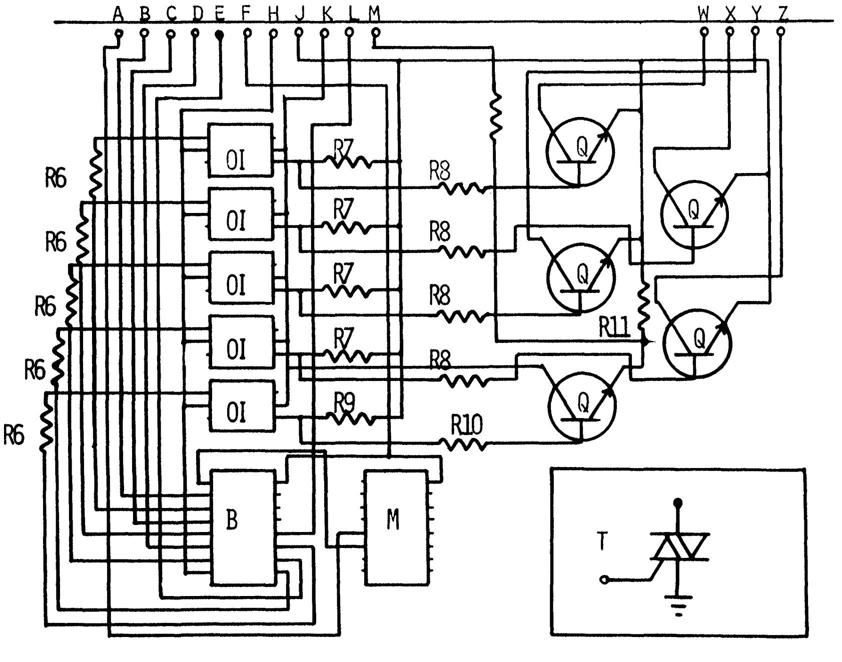

*Figure 3. Motor control R6 = 0.1K$\Omega$; R7 = 1.0K$\Omega$; R8 = 0.68K$\Omega$; R10 = 1K$\Omega$; R11 = 220$\Omega$; 01 = FDC 860 optolsolators(Fairchild); B = DM80C97 hextristate buffer(National Semiconductor); C = 9602PC dual monostable multivibrator-(Fairchild); Q = TiP29 Power transistors(Fairchild); M = 9602PC dual retriggerable resettable monostable multivibrator (Fairchild); T = Triac (Archer Electronic Parts); E = 22 pin edge connector (a, +12 V; b, power gnd; c, cpu gnd; d, +5 V; e,f,h,j, microprocessor I/O, PA4-7; R, CB2; I, PA3; M, output to triac).*

attached to an aluminum mount which is free to move along two aluminum rods lubricated with graphite and attached to the base plate.

The syringe burette delivers titrant through a louer fitting on a syringe needle and Teflon tubing. The end of the tubing is heated and flattened to provide a bladder-type valve at the end. This design minimizes solution egress due to gravity when not being driven by the stepper motor. One milliliter is delivered with a series of 1200 pulses to the motor, which yields precision for solution delivery of one part out of 1200, or 0.08%. The reaction vessel is thermostated to ±0.1 °C for thermal stability and is covered with a Plexiglas cap drilled with appropriate holes for electrodes, thermometers, titrant delivery tubes, and tubes for nitrogen purging.

### Experimentation

To demonstrate the use of the titrator, a strong acid-strong base titration was conducted. The potentials of the pH = 4.008 phthalate and the pH = 6.865 phosphate buffers were recorded until stable voltages were obtained. Then 100.0 ml of 0.03819 M HCl was pipetted into a reaction vessel thermostated at 25.0 °C, and the syringe burette was filled with 0.2550 M NaOH. The parameters chosen for the titration were constant 0.050 milliliters titrant increments, a stability criteria of 0.005 V maximum change between sequential readings of the same titration point, and 4 min between readings of the electrode. The titration was automatically terminated when the delivered volume of titrant exceeded 1.85 milliliters (see figure 5).

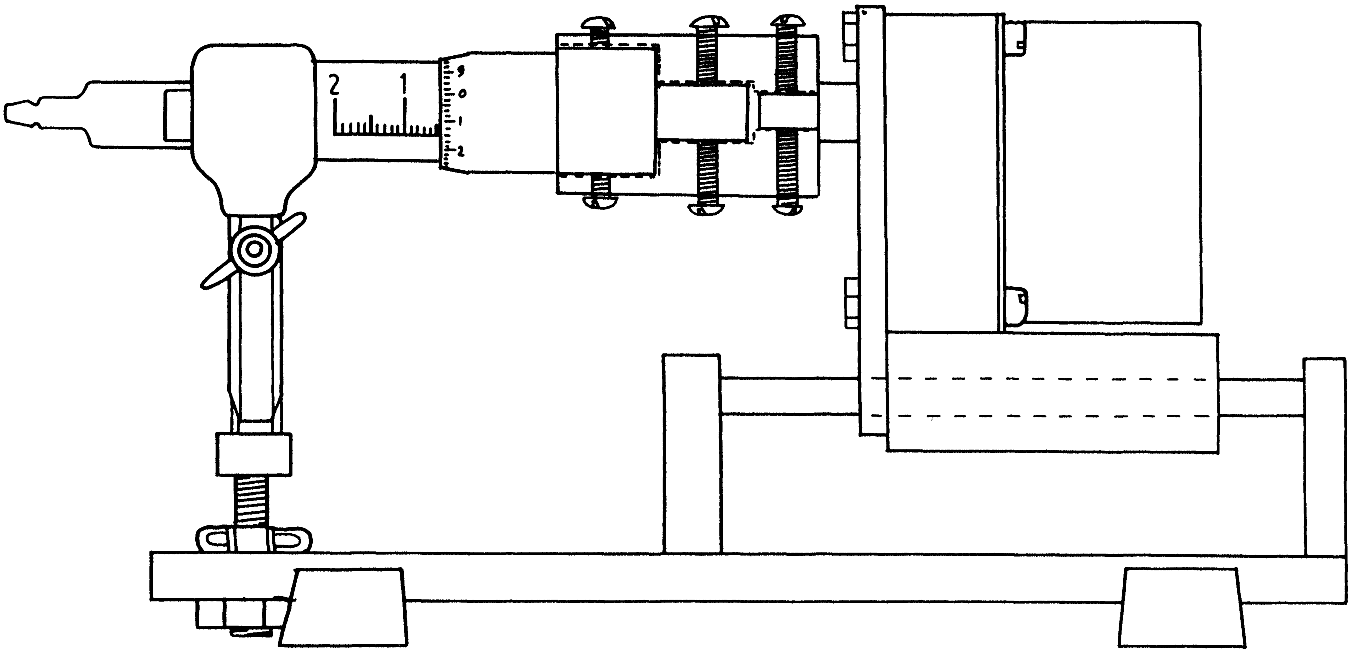

*Figure 4. Stepper motor and syringe mount. The stepper motor is model K82821-P2 (North American Philips Controls, Corporation), reversible unipolar with a 10 : 1 reduction. The syringe is 2.0 ml with a luer connection (Gilmont Industries, Inc).*

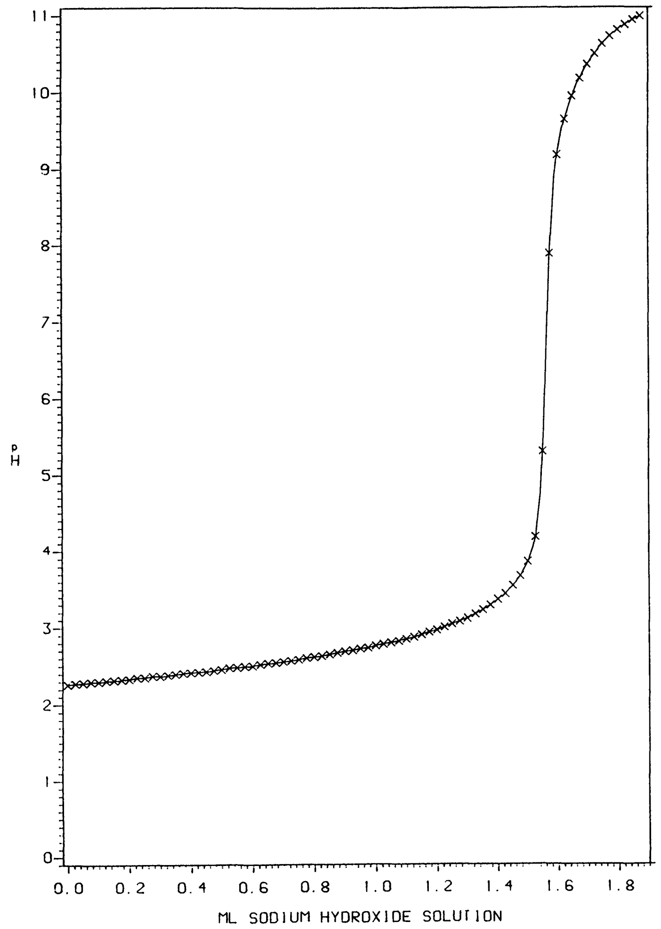

*Figure 5. Titration of 0.03819 M HCl with 0.2550 M NaOH with equal volume addition.*

The voltage measurements were displayed on the monitor during the titration, output to the printer, and at the end of the titration stored on a disk file. A second program opened this file and converted the voltages to pH values using the potentials of the buffers initially measured. The data was output serially to a Data General Eclipse S/130 minicomputer. In the word-processor the data file was converted into a program file and submitted to the University of Rhode Island's mainframe computer for graphic display by means of SAS Graphic routines [21].

The system has been in use in our laboratory for over a year and has been invaluable in conducting a number of types of titrations. The modular programming style has allowed easy modification of software to use the system to its full advantage. Data storage on floppy disk and magnetic tape on the Eclipse system has provided efficient cataloguing of experimental records. Final publishable quality graphs are typically available 15 min after experimentation.

### References

1. Bjerrum, J., *Amine Formation in Aequous Solutions* (P. Haase and Son, Copenhagen, 1941).
2. Jagner, D., *Analytica Chimica Acta*, **50** (1970), 15.
3. Anfalt, T. and Jagner, D., *Analytica Chimica Acta*, **57** (1971), 177.
4. Frazer, J. W., Kray, A. M., Selig, W. and Lim, R., *Analytical Chemistry*, **47** (1975), 869.
5. Ariano, J. M. and Gutknecht, W. F., *Analytical Chemistry*, **48** (1976), 281.
6. Christiansen, T. F., Busch, J. E. and Krogh, S. C., *Analytical Chemistry*, **48** (1976), 1051.
7. Leggett, D. J., *Analytical Chemistry*, **50** (1978), 718.
8. Wu, A. H. B. and Malmstadt, H. V., *Analytical Chemistry*, **50** (1978), 2090.
9. McClintock, S. A. and Purdy, W. E., *Analytical Letters*, **15** (1982), 1001.
10. Ben-Yaakov, S., Raviv, R., Guterman, H., Dyan, A. and Lazar, B., *Talanta*, **29**, (1982), 267.
11. Gran, G., *Acta Chemica Scandia*, **4** (1950), 559.
12. Gran, G., *Talanta*, **77** (1952), 661.
13. Ingman, F. and Still, E., *Talanta*, **13** (1966), 1431.
14. Johansson, A. and Pehrsson, L., *Analyst*, **95** (1970), 535.
15. Gans, P., *Advances in Molecular Relaxation and Interaction Processes*, **8** (1980), 139.
16. Zuberbuhler, A. D. and Kaden, T. A., *Talanta*, **29** (1982), 201.
17. May, P. M., Williams, D. R., Linder, P. W. and Torrington, R. G., *Talanta*, **29** (1982), 249.
18. Forcé, R. K., Boyd, J. and Harris, E., *Interfaces in Computing*, **1** (1982), 59.
19. Migneault, D. R., Mattera, R. and Forcé, R. K., *Journal of Automatic Chemistry*, **8** (1986), 32.
20. Migneault, D. R. and Forcé, R. K., *Analytical Chemistry*, **54** (1982), 2628.
21. SAS Institute, Inc., in *SAS/Graph User's Guide* (SAS Institute, Inc., Box 8000, Cary, North Carolina 27511, USA, 1981), 69.

